# Radiography managers’ perceptions on skills required in public health institutes in Gauteng

**DOI:** 10.4102/hsag.v29i0.2654

**Published:** 2024-08-30

**Authors:** Joseph L. Mopeli, Portia N. Ramashia, Lynne J. Hazell

**Affiliations:** 1 Department of Medical Imaging & Radiation Sciences, Faculty of Health Sciences, University of Johannesburg, Johannesburg, South Africa

**Keywords:** radiography, radiography managers, perceptions, coaching, mentoring, public health institutions

## Abstract

**Background:**

Management of radiography departments requires skilled and competent managers. This task becomes complex if there is no management development and collaborative performance monitoring.

**Aim:**

The study aimed to explore and describe the radiography managers’ perceptions regarding management training and skills required.

**Setting:**

The research was conducted in public health institutions of Gauteng, South Africa.

**Methods:**

Qualitative, exploratory and descriptive approach was adopted, and a purposive sampling method was used to select twenty-three (23) managers from the radiography departments; however, data saturation guided the sample size. Data were collected through online focus group discussions (FGDs). Ethical approval was obtained through Departmental Research Committee (DRC) of Medical Imaging and Radiation Sciences (MIRS) department, Higher Degrees Committee (HDC), Research Ethics Committee and Gauteng Department of Health Research Committees. Data trustworthiness was obtained through member checking, data verification and an independent coder to verify the accuracy of the data. Thematic data analysis method was used to analyse the data.

**Results:**

Five themes emerged from the thematic analysis and centred on: difficulties in transitioning into management, lack of management support, the need for postgraduate management qualification, coaching and mentoring and required skills for radiography managers.

**Conclusion:**

Public health institutions continuously face transitions in service delivery frameworks. This requires competent and skilled radiography managers to survive in this environment. The study revealed that new managers experience difficulties and require management support to succeed in their roles.

**Contribution:**

Awareness of managers developmental needs relative to the real-life dynamics of radiography management in Gauteng public health environment.

## Introduction

Globally, effective and efficient management plays a critical role in the success of public healthcare service delivery in developing countries such as South Africa (Mukwakungu, Mabasa & Mbohwa 2018; Srivastava & Kunwar 2018; Teame, Debie & Tullu 2022). South Africa (SA) has been striving for an all-inclusive public service; however, leadership still stands as a challenge in the healthcare sector (Mukwakungu et al. 2018), and this warrants the developing discourses on leadership scrutiny and improvement (Klingborg, More & Varea-Hammond 2006). In Canada, Plecas, Squires and Goris (2018) add that as much as there is abundance of leadership discourses and models, there are still considerable gaps across public service organisations. While the SA government appears to have made strides in transforming the public healthcare system, through new policies such as the *National Health Act 61 of 2023*, there are persisting challenges. These challenges include dilapidating infrastructure, resources shortages and public dissatisfaction (Maphumulo & Bhengu 2018). In a study by Malakoane, Heunis, Chikobvu, Kigozi and Kruger (2020) in the Free State Province, it is asserted that failure to implement health policies and align resources allocation to demand is attributed to poor governance and leadership. In SA, the public service institutions are often viewed as unethical, inept and led by incompetent leaders that deliver substandard services to the public (Motshwane 2018; Sebola 2018). The performance of healthcare institutions and managers requires contextual monitoring and development to ensure transformation of the system (Shai & Ogunnubi 2018).

There is a perception that to survive in the new public service realm wherein, generally, the needs of society play a key role, employment of capable, skilled and competent leaders to promote organisational efficiency and public trust is crucial. (Gamage 2014). In this light, there is a need for continuous collaborations that promote empowerment of radiography managers through programmes such as coaching to ensure excellent quality and impactful service delivery.

The researcher is employed as a senior manager at the Gauteng provincial Head Office and is responsible for providing strategic guidance and support to the radiography managers in all Gauteng Department of Health (GDoH) institutions and has observed considerable scarcity of management skills development avenues for newly appointed radiography managers. Furthermore, the researcher had recently conducted a research study to investigate the required management training, skills and competencies required by the managers for them to perform optimally in their roles. This study revealed that there is a need for focused programmes that will facilitate management development and support for the radiography managers to enable them to perform optimally in their roles. Absence of good leadership in the public healthcare environment can be detrimental to the ability of the healthcare institutions to deliver high-quality and impactful healthcare outcomes. The Radiography department is part of a health institution responsible for the provision of radiography services (Health Professions Council of South Africa [HPCSA] 2023) and similarly to other health departments, comprises of financial, human and physical resources that require sustainable coordination and utilisation (Daweti & Evans 2017).

The aim of this research study was to explore and describe the perceptions of the radiography managers on the management training and skills required to succeed in their roles. The focus will address the five themes and subcategories that emerged from the research.

## Research methods and design

A qualitative, exploratory and descriptive design was adopted for this study to explore the real-life experiences of the radiography managers. This approach was deemed suitable as it provided an opportunity for the researcher to gain an in-depth understanding of the radiography managers individual perceptions of the management skills and competencies required in the public institutions in Gauteng (SA) province after their appointment as managers. Data collection was conducted through online focus group discussions (FGDs).

### Research setting

The study was conducted at the GDoH institutions in South Africa. The GDoH currently has a total of 52 Radiography departments, across 5 districts, that have officially appointed managers responsible for strategic management and leadership while ensuring delivery of quality services to the community (GDoH 2019:37). The Radiography departments are spread across all five Health Districts of the province, namely, City of Johannesburg, West Rand, Tshwane, Sedibeng and Ekurhuleni Districts (GDoH 2019).

### Population and sampling

A purposive sampling technique was used for this study. The use of this method afforded the researcher to select information-rich participants who are employed as managers in the public health institutions in Gauteng, South Africa (Nyumba et al. 2017). At the time of the research, the total population of radiography managers in GDoH was 35. A total of five FGDs were conducted with the total number of 23 participants. All radiography managers were invited to participate in the study; however, only 23 consented to participate in the study. Each focus group consisted of an average of five participants. The focus groups included Chief Radiographers, Assistant Directors and Deputy Directors. The inclusion criteria required that the sample population comprise radiographers who were managers of Radiography imaging and radiation therapy departments across GDoH public institutions and registered with the HPCSA as an independent practitioner. The sample size was determined once there was no new data emerging from the FGDs (Hennink, Kaiser & Weber 2019). The participants who were deemed suitable were selected from the GDoH’s radiography departments’ managers database. At the time of this research study, all the departments had appointed managers who were responsible for ensuring strategic and clinical leadership in the departments. The Radiography departments include the categories: Diagnostic, Ultrasound, Nuclear Medicine and Radiation Therapy. The management positions in the Gauteng Department of Health vary according to the category or level of institution where one is employed. As a result, Radiography management echelon include Deputy Directors (DD), Assistant Directors (ASD) and Chief Radiographers (CR). The Deputy Directors are employed in central hospitals, Assistant Directors are employed at central, tertiary, regional and specialised hospitals and the Chief Radiographers are employed at the community health centres (CHCs) and district hospitals. The radiography managers, across different levels of employment, play a similar role in ensuring that the departments are effectively managed and department’s strategic objectives are met. The differences in the level of employment or institution, either tertiary or district, do not warrant limitations on management activities, duties and performance indicators from the radiography managers.

### Data collection

Data collection was conducted through semi-structured online FGDs, using Microsoft Teams, which permitted a variety of responses to prearranged questions and provided the researcher with detailed responses (Ugwu & Eze Val 2023). The interviews included open-ended questions about the perceptions of the managers on required management skills and competencies. The FGDs were conducted by the researcher who assumed the moderator’s role to ensure full participation of all the participants in the discussions.

A total of five FGD sessions took place from May 2022 to June 2022. Each FGD was set to last an average of 1 h and 30 min with an average of four participants to ensure all participants’ inclusion and understanding of the questions; the FGD sessions were conducted in English language and all the participants were comfortable with the English language. The non-verbal cues, such as body language, could not be observed as the sessions were conducted online, and the cameras were switched off throughout all the sessions because of poor connectivity that results when all participants have their cameras switched on.

### Data analysis

Thematic data analysis approach rendered the researcher ability to identify, create and interpret patterns in the data collected (Proudfoot 2023:308). Moreover, an independent and knowledgeable coder was used to enhance the researcher’s reliability (Morgan 2022). The themes were generated using the six-step approach identified by Braun and Clarke (2006:16–23), which requires the researcher to engage in familiarisation with the data through transcription and repetitive reading of the data to gain a deeper understanding of the participants’ experiences, generating initial codes through identifying keywords and expressions, searching for the themes by collaborating codes that related to common ideas, reviewing the themes to ascertain coherence and alignment to the research questions, defining and naming the themes by creating definitions for each theme and lastly producing the report that produced a narrative that defines the themes as pertaining to the participants’ perceptions.

### Measures of trustworthiness

This refers to the findings of the study being based on the discovery of human experience as it was experienced and observed by the participants. Trustworthiness of this study was attained through the use of the four trustworthiness criteria proposed by Lincoln and Guba (1985) including credibility, transferability, confirmability, dependability, triangulation and using the independent coder (McGrath, Palmgren & Liljedahl 2019). To ensure credibility of the findings and reduce potential researcher’s bias creep, an independent coder was used.

To ensure transferability, the full details and description of the participants’ selection criteria and sampling approach were provided (Stahl & King 2020). Furthermore, the description of the setting for the FGDs is also provided to facilitate adaptation of the shared experience (Birt et al. 2016). Confirmability centres on the extent to which collected data and findings can be confirmed by different researchers (Korstjens & Moser 2018). In this study, confirmability was achieved through an audit of data collected and accuracy of the raw data. The researcher used a log to document impulsive notes about the FDG sessions and key aspects of the FGDs identified throughout the sessions as part of the data quality audit (Kim et al. 2020).

Member checking and triangulation were conducted to sustain the consistency of the findings (Nowell, Norris & White 2017). Participants were asked to verify the researcher’s interpretation of what they meant and this was done after transcription and analysis of the discussions during all FGDs as suggested by McGrath et al. (2018). To avoid bias, field notes, reflective journal and audio recordings confirmed the facts emanating from data. Furthermore, the process of reflexivity to assess own biases, preferences and preconceptions was used by the researcher.

### Ethical considerations

The Health Research Ethical Committee at the University of Johannesburg (REC-1258-2021) granted clearance for the study’s ethical compliance. The Chief Executive Officers (CEOs) and delegated research committees from the institutions under the research gave permission to conduct the study using the radiography managers as the participants. The consent to participate in the FGD interviews and to audio-record the discussions was also obtained before the FGD sessions. Participants were also informed of their freedom to withdraw from the study at any time. The audio-recording and transcripts that were used for data analysis are password protected. The data files are to be deleted after 5 years.

## Findings and discussion

The study participants were managers employed in the diagnostic, radiation therapy and nuclear medicine disciplines. The sample included 4 male and 19 female participants. Thirteen participants were Assistant Directors, and ten participants were Chief radiographers who worked as managers of respective departments within the public hospitals and health districts in Gauteng province. All the participants were radiographers registered with the HPCSA. The qualifications of the participants included National Diploma, Bachelor in Radiography and post-graduate qualifications in management. The age of the participants ranged between 30 years and 63 years.

Five main themes and seven sub-themes emerged from the FGDs relative to the radiography managers’ perceptions on the management training and skills required in the public health institutions of GDoH as depicted in Table 1:

**TABLE 1:** Generated themes and sub-themes.

Themes		Sub-themes
1.	Transitioning into radiography management is a complex and challenging process	1.1 Absence of structured orientation and induction for radiography managers 1.2 Openness about the job expectations of radiography managers
2.	Lack of management support for the newly employed radiography managers	2.1 Absence of on-boarding programmes focused on radiography managers 2.2 Lack of empowerment strategies for the subordinate radiography staff
3.	Need for postgraduate management qualification	3.1 Absence of management qualification
4.	Coaching and mentoring for radiography managers	4.1 The need for coaching and mentoring for radiography managers
5.	Management competencies and skills for radiography managers	5.1 The need to acquire new management skills and competencies

### Theme 1: Transitioning into radiography management is a complex and challenging process

The aim of this research was to explore and describe the perceptions of the radiography managers employed in the Gauteng public health institutions on management training and skills required. This theme refers to transitioning into radiography management as a complex and challenging process. Mallaby, Prince and Hofmeyer (2017) and Roberston (2020) assert that the management development and learning process are among factors that contribute to the difficulties in transitioning, and these factors may differ on an individual basis and successful transitioning to the management role entails value adjustment and transformation. The findings regarding the complexities and challenges in transitioning into radiography management are presented in the two subthemes discussed further in the text.

#### Sub-theme 1.1: Absence of structured orientation and induction for radiography managers

The radiography managers form part of crucial manpower in the healthcare matrix and often play a gate-keeping role in ensuring that the healthcare organisations render sustainable services to the public. Therefore, similar to other managers, radiography managers experience difficulties when they switch from a production to a management position and require support and unceasing development such as structured orientation and induction (Nkwana 2014). Orientation, induction and onboarding are programmes that are planned to provide new employees with the required skills, tools and knowledge to operate efficiently and independently in the organisation (Raj 2017). These programmes also introduce a new network of events and organisational processes to the new manager in order to assist them in succeeding in their position (Takeuchi, Takeuchi & Jung 2021).

The participants, however, expressed their concerns about the lack of structured orientation and induction programmes tailored for radiography managers as illustrated in the quotations listed further in the text:

‘For me, it was very difficult to transition from being a Chief Radiographer to being an Assistant Director… the staff treat you differently and have new different demands and the senior management and CEO were not impressed with me …’ (Female, 42 years old, Assistant director)

‘I had no full support, but had to use experience from managing smaller units from previous employment …’ (Male, 34 years old, Assistant director)

‘[*M*]y experience was not very bad because I knew what was expected and I had no expectations of being mentored into the post.’ (Female, 40 years old, Assistant director)

‘[*T*]he transition was difficult at the beginning as support from the Head of Department was limited.’ (Female, 49 years old, Assistant director)

The responses indicated that the majority of the managers experienced challenges in their transition process and expressed that the process of transitioning into a new management role could have been made smooth by the implementation of orientation and induction for new managers (Mallaby et al. 2017). GDoH sources training opportunities for various management levels from the Gauteng City Region Academy (GCRA) and the National School of Government (NSG) (GDoH 2020). In contrast, most of the managers expressed that they received no structured onboarding, induction or orientation about the job demands and expectations. Some participants reported that their previous experiences as operational supervisors assisted them in their transition to their management roles.

#### Sub-theme 1.2: Openness about the job expectations of radiography managers

The participants highlighted that communication about the job expectations from the seniors was not clear. The participants further expressed that their new responsibilities included solving problems and to collaborate, which requires shared understanding and perception about the tasks and expected outcomes. They further mentioned that clear job descriptions and requirements would assist in comprehending their roles better. Participants’ responses reflected that they did not receive adequate guidance on what the top management expected from them. The participants’ responses included the following:

‘[*T*]he previous manager who occupied the position was not approachable and could not learn from them. When I got the position, the Medical Superintendent was not helpful as they had no knowledge about the department and my job.’ (Female, 42 years old, Assistant director)

‘[*A*]s a Chief Radiographer, you report to the Clinical Manager who is a doctor who sees over other doctors and staff. They have absolutely no clue about anything in Radiography. So, you need to source your own things together, type your things, whatever you want, explain to them what it is and then just ask him to sign. But they don’t have a clue of Radiography, so there is no support type of things from the top.’ (Female, 50 years old, Chief Radiographer)

‘[*T*]here is a need for people to know what to expect before they start the position.’ (Male, 38 years old, Assistant director)

‘… I felt that I was thrown in the deep end and I needed a protocol on how an AD should work.’ (Male, 44 years old, Deputy director)

Moreover, identifying the misalignment and lack of universality of the job descriptions among managers could be contributing to the reason for managers struggling with the transition to their new management roles. To bridge the gaps in how different managers perceive their roles, it is essential that new radiography managers be exposed to skills building and coaching programmes to provide the new managers with the relevant coping mechanisms. The induction, orientation and informal training will assist new managers to easily integrate into the organisation and gain a practical understanding of the organisation and its systems. Executive management should ensure that they create opportunities for open and supportive learning to help new employees to be innovative and proactive in dealing with service delivery dilemmas within their area of management. Participants reported that they often found themselves short of skills that could help them deal with the transition phase. In addition, some participants reported that although they possessed the academic management education and qualifications, they still required practical tact on how to blend and apply their academic knowledge in real-life circumstances.

### Theme 2: Lack of management support for the newly employed radiography managers

This theme discusses the lack of supervisor’s support for the radiography managers. Management support is defined as the attributes, positive attitude and behaviour of the organisational executive team towards a work mission throughout its lifespan (Kemei, Oboko & Kidombo 2018). The attitude and support of the executive management are key catalysts towards the success of the organisation and have a direct link to the level of organisational performance (Schachtebeck & Niewenhuizen 2015). Management support can be realised in the form of direct or indirect coaching of employees. The objective of this is to facilitate empowerment and motivation for employees.

The participants in this study conveyed diverse experiences regarding the level of support they received from their top management and how it aided their ability to survive. Participants expressed the essence of receiving management support and that it played a role in assisting them to transition to their new position and performing optimally. These findings are dissected further into the sub-themes discussed further in the text.

#### Sub-theme 2.1: Absence of on-boarding programmes focused on radiography managers

All the participants have, at some point in their profession, been exposed to being new managers of different departments in the public service. From the participants’ narratives, factors that influenced the different experiences included the presence of hand-over systems, previous experience and orientation into new environment. Participants’ responses showed that a system of familiarising them with the internal business processes and expectations of the job was needed. The quotes discussed further in the text emphasise this:

‘[*E*]very place has a way of doing things and that necessitates the on the job coaching in order to learn and understand the organisational culture and internal processes.’ (Male, 34 years old, Assistant director)

‘[*E*]very organisation has a way of doing things and you have to be given a guide on how to do certain things.’ (Female, 57 years old, Chief Radiographer)

The central idea narrated by all participants was that being acquainted with the working environment and the internal procedures is critical. Therefore, they had the expectation of being provided with a clear hand-over document, standard operations procedure or job description. This deduction is confirmed in the following quote:

‘When I was appointed as a manager, I had no management training or mentoring given to me. The expectations were just put on me and no methodology of how to address them was given …’ (Male, 39 years old, Assistant director)

Some participants expressed that they received hand-over and indicated that the support enabled them to perform better and educate others about their role in the institution. This is reflected in the quotation below:

‘I had great support from the facility manager and Head Office. Some managers sometimes do not understand the role of radiography managers or Assistant Director as it was a new position and I had to educate others about this new role …’ (Female, 39 years old, Assistant director)

However, some participants reported that some managers enforce that their own ideas must be used in the management of the department, and they do not regard the ideas and traits of the newly appointed radiography managers. They explained that it is important that as although they are new, they should be afforded the space to perform and try their own models:

‘… I received the support from the manager, however, it becomes a problem if you have to copy the mentor. You need the space to be innovative and apply your knowledge.’ (Female, 50 years old, Chief Radiographer)

‘I received support from the facility manager and the Assistant Director of the programme …’ (Female, 29 years old, Chief Radiographer)

Suknunan and Bhana (2022) claim that a positive and supportive relationship between an employee and a manager is linked to motivated and enhanced performance. Findings of the current study suggest that in some institutions, radiography managers have an executive team that has structured support systems and processes. However, there were some institutions wherein radiography managers had no direct or formally structured support systems. This could be attributed to a lack of understanding and integration of the radiography programme into the holistic healthcare support services framework in some institutions. Other managers stated that they did not receive adequate support and hand-over from their managers and had to rely on colleagues from other institutions for information. Some of the participants’ responses are presented further in the text:

‘I think mentoring was going to help a lot because sometimes you are being given a job and given an office, being told that you’re going to be running this department but not being given transparently what are they expecting from you. You come and manage this department. With me I experienced a lot of challenges …’ (Female, 58 years old, Assistant director)

‘… I was from a different province so there were a lot of things that I needed to familiarize myself with so I had to ask from all the other chiefs that have been here just for advice.’ (Female, 29 years old, Chief Radiographer)

Chinn (2017) adds that a good hand-over helps new staff members to settle into their new position confidently and management support is not limited to the provision of soft skills such as training but includes the provision of physical resources or tools to enable new managers to perform the expected duties (King’oo 2017).

#### Sub-theme 2.2: Lack of empowerment strategies for the subordinate radiography staff

This sub-theme discusses the need for radiography managers to have strategies in place to empower their subordinate staff, which can lead to better outcomes for the department. The participants mentioned that as much as formal qualifications prepare one for the management role, being exposed to development platforms, prior or after appointment into the management position assists in coping with anxieties. Few participants in the current study conveyed that being given the opportunity to partake in the platforms that entrench empowerment skills assisted them in coping with the requirements of their new role. On the contrary, some managers shared that the absence of management development platforms and lack of previous management experience contributed to their diminished ability and competency to derive solutions for the dilemmas that came with their new jobs. This notion is evidenced in the responses listed further in the text:

‘[*M*]y role as a team leader from my previous position provided me with experience for the new post. But there is a need to for one to travel the management journey and acquire relevant knowledge before they enter the management role.’ (Female, 39 years old, Chief Radiographer)

‘I had a Bachelor of Technology [*BTech*] degree but did not really help me with the new job requirements but experience from the other junior positions assisted me …’ (Female, 54 years old, Assistant Director)

Employee empowerment is one of the most efficient methods for enhancing employee performance and ability to succeed. Empowerment is a process through which the organisation develops the ability to improve performance and forms part of the development strategy for employees (Ivanova & Von Scheve 2019). The employees’ morale, creativity and organisational effectiveness are enhanced when they are given opportunities and job tasks that empower them. Vu (2020), adds that empowering leadership or those in management encourages employees to perform better because of the power that they are given. Empowering employees translates to strong professional relationships that are based on mutual trust, organisational values and shared perceptions about job expectations (Kumar & Kumar 2017). Executive management support involves setting up an effective system and providing relevant support for work activities to achieve work objectives (Kemei et al. 2018). Radiography managers and other levels of professionals must be included in the cohort of the beneficiaries of the available management support avenues, such as coaching and mentoring within the institution.

### Theme 3: Need for postgraduate management qualification

The discussion on this theme is centred around the educational background that participants possessed when they were appointed into the management position. The special area of inquiry was the management education that is offered at the postgraduate level. Management education is faced with complex challenges that are attributed to the fast-paced changes that constantly occur in most industries (Tienhaara, Lyytinen & Kivisto 2016).

#### Sub-theme 3.1: Absence of management qualification

This sub-theme relates to the lack of formal management qualifications among radiography managers, which can impact their ability to perform their role effectively. Participants expressed that for them to achieve their management objectives, they needed to possess general management qualifications and must have participated in a management programme that focuses on aspects of the public sector such as policies and service delivery models used in the public sector. Participants similarly articulated that being in possession of a postgraduate management qualification plays an important role in preparing one for the management role. However, it needs to be accompanied by experiential learning. The perceptions of the participants are included in the responses listed further in the text:

‘I did not have any management qualification when I got appointed and I am not sure if it would have helped me fully. But there is a need for public sector management training for new managers as this would help with understanding the environment …’ (Female, 42 years old, Assistant Director)

‘I had a postgraduate diploma in management and it helped me with building a foundation for the management expectations …’ (Female, 40 years old, Chief Radiographer)

‘I had a master’s degree and it helped prepare me in my position as an Assistant Director [*AD*] and boosted my confidence …’ (Male, 39 years old, Assistant Director)

‘I had a Bachelor of Technology [*BTech*] degree, but, it did not offer full management training but it taught me operational management aspects. I am currently pursuing a Postgraduate diploma in Public Health and it is contributing to my understanding the job better …’ (Female, 31 years old, Chief Radiographer)

Possessing management education qualification has significant benefits such as career advancement, acquiring specialised knowledge and enhancing professional skills (Daweti & Evans 2017). However, Nkwana (2014) notes that the development of leadership capacity within a work context and an organisation is still required to enable employees to achieve set objectives. However, the complexity of the management education programmes is also influenced by changes in the industry (Tiehaara, Lyytinen & Kivistoo 2016). Globalisation of management practices in sectors that provide public services emphasises the need for higher education institutions to reform their programmes in a way that continuously responds to the needs of the public service industry.

### Theme 4: Coaching and mentoring for radiography managers

This theme refers to the need for coaching and mentoring programmes for radiographers once they are appointed into the management role. Coaching and mentoring are management activities that facilitate the learning of new skills to nurture staff performance and deliver organisational results (Suknunan & Bhana 2022). They are, fundamentally, learning and development activities that share similar roots (Serrat & Serrat 2017). Coaching and mentoring, as a leadership support and development mechanism, require adequate understanding of the organisational culture, needs and processes (Al Hilali, Mughairi, Kiand & Karim 2020).

Coaching and mentoring programmes have been identified as a need for radiography managers to assist them to develop the required competencies and skills to address the challenges faced within their leadership tenure (Kester 2017). Coaching and mentoring entrench new skills and capabilities for one to be able to independently address challenges in the workplace (Serrat & Serrat 2017). There is no single theory agreed upon that can address all leadership development challenges. Mvelase (2019) argues that coaching and mentoring have gained popularity as a progressive leadership development tool in South Africa. Several theories have been developed to facilitate effective coaching and mentoring. Almost all the theories are centred around the achievement of set goals and the development of the coachee (Blackbyrn 2022; Kunos 2019; Wilson 2017).

Multiple proponents of coaching and mentoring identify the T-GROW theory, a four-step process that focuses on theme or topic, goal, reality, options and willingness, as the most suitable approach when targeting adult participants and focus on self-realisation and growth of others (binti Kamarudin, binti Kamarudin, binti Darmi and binti Saad 2020); Kunos 2019; Thipatdee 2019). An alternative theory is the adult learning approach that stimulates self-reflection, deep and experiential learning. Blackbyrn (2022) maintains that adults learn best by reflecting on past experiences when solving dilemmas. Arghode, Brieger and McLean (2017) propose that adult learning usually requires little help from instructors but rather requires more focused support and direction. Adult learners often require immediate application of learned knowledge as they learn best when they choose the content and context.

The dilemma of the lack of leadership support structures for public healthcare managers is not only pronounced in South Africa but rather a challenge for most developing countries (Khan, Nawaz & Khan 2016). Lack of good leadership and its development exposes the organisation to a range of risks such as failure to deliver effective healthcare outcomes. The managers who are expected to execute the mandate are more likely to miss the opportunities to create collaborative alliances and relationships (Khan et al. 2016). On service delivery outcomes, poor leadership leads to poor quality and misaligned service delivery, which are characterised by high mortality rates, increased litigations, high disease burden and poor access to services by the citizens (Srivastava & Kunwar 2018).

Higher education institutions in SA that provide radiography training offer basic management principles within their curriculum, however, do not extensively focus on personal skills. As a result of the lack of focused leadership training, leadership challenges may be observed during the tenure of radiography managers. As much as there are various management courses offered to all public officials through various platforms such as internal training partnerships and the National School of Governance (NSG), there are no coaching and mentoring programmes that mandatorily focus on the radiography managers. Kester (2017) adds that coaching and mentoring play a key role in preparing the managers for their roles and are likely to facilitate easy adaptation. The radiography managers are adults expected to possess knowledge above other staff members and lead them towards high performance levels.

The findings regarding coaching and mentoring for radiography managers are presented in the sub-theme listed further in the text.

#### Sub-theme 4.1: The need for coaching and mentoring for new radiography managers

Radiography managers are a cohort of healthcare workforce that plays a crucial role in ensuring that the healthcare institution provides a comprehensive diagnostic service package using continually evolving technological and environmental advancements. To achieve this mandate, the radiography managers similarly to other managers, require continuous development, such as coaching and mentoring, within the work context (Nkwana 2014) and in alignment to the changes in the related industry (Tiehaara, Lyytinen & Kivistoo 2016). Coaching and mentoring refer to management activities that enable and teach new skills to nurture one’s performance for personal and organisational goals (Serrat 2017; Suknunan & Bhana 2022). Coaching and mentoring support learning and gaining knowledge about the institution and promote the culture of developing new concepts and ways of doing the work (Hollywood et al. 2016). In response to the question, the participants conveyed their concerns about the limited availability or absence of structured coaching and mentoring programmes as exhibited by the following quotations:

‘[*N*]o mentoring was received from the Clinical Manager as they have no knowledge of Radiography …’ (Female, 50 years old, Chief Radiographer)

‘[*N*]o coaching was received internally. You are mostly on your own and have to look for most tools on your own …’ (Female, 40 years old, Assistant director)

‘[*E*]ducation is not something that should be acquired once off, in 2010 you qualified to get a qualification. Because things change all the time, I would I personally like to continuously be gaining, upgrade and adapting to an everchanging work environment.’ (Female, 39 years old, Chief Radiographer)

The responses provided a view that some managers received coaching of some kind when they were appointed into their management roles, while others expressed that they had received no coaching or mentoring when they were appointed. Participants expressed that although they received some coaching from their managers, they still had challenges with its adequacy. Participants conveyed their reflections in the citations provided further in the text:

‘… I received the support from the manager, however, it becomes a problem if you have to copy the mentor. You need the space to be innovative and apply your knowledge …’ (Female, 50 years old, Chief Radiographer)

‘I had great support from the facility manager and Head Office. Some managers sometimes do not understand the role of radiography managers or Assistant Director as it was a new position and I had to educate others about this new role …’ (Female, 39 years old, Assistant director)

‘… I know that I have a coach. I did not go through any training but as time goes on I learned from my AD and I learn how to manage and how to deal with certain issues …’ (Female, 29 years old, Chief Radiographer)

The participants raised concerns about the risks of their poor or misaligned performance, which may lead to unsustainable and poor quality services to the clients. The participants further expressed that coaching and mentoring would enhance their awareness about the culture of the organisation and help them learn about the standards or procedures followed to achieve certain departmental objectives:

‘… I think mentoring was going to help a lot because sometimes you are being given a job and given an office, being told that you’re going to be running this department but not being given transparently what are they expecting from you. You come and manage this department. With me I experienced a lot of challenges …’ (Female, 58 years old, Assistant director)

‘[*E*]very place has a way of doing things and that necessitates the on the job coaching in order to learn and understand the organisational culture and internal processes …’ Male, 34 years old, Assistant director)

‘[*E*]very organisation has a way of doing things and you have to be given a guide on how to do certain things …’ (Female, 57 years old, Chief Radiographer)

Identifying the absence of structured on-the-job training programmes could be the contributing factor as to why the participants receive limited or no coaching and mentoring. The executive management needs to spend more time with the newly appointed radiography managers to understand their development needs as this could aid in coaching and mentoring sessions that address the expected performance outcomes and standards. The dilemma of the lack of leadership support and management development structures for public healthcare managers is not only pronounced in South Africa but for most developing countries (Khan et al, 2016). Lack of good leadership and management practices often expose the organisation to a range of risks such as failure to deliver effective healthcare outcomes. Khan et al. (2016) further posit that managers who are expected to execute the management duties, if not continually developed, are more likely to miss the opportunities to create collaborative alliances and relationships that can offer better solutions to service delivery challenges. Hollywood et al. (2016) further report that coaching and mentoring support personal learning and innovative thinking that can benefit the public healthcare radiography management fraternity.

### Theme 5: Management competencies and skills required for radiography managers

This theme represents the need for radiography managers to acquire new management skills and competencies. Appointment as a manager undoubtedly brings joy and some sense of great accomplishment to any professional; however, the role also brings the need to perform introspection and audit of skills that one possesses to establish their suitability for the new role:

‘The same way we expect production radiographers to participate in continuous professional development. I think we as managers as well there should be some sort of programs for us to continue developing ourselves at that level as well …’ (Female, 29 years old, Chief Radiographer)

#### Sub-theme 5.1: The need to acquire new management skills and competencies

This sub-theme relates to the need for radiography managers to develop their skills and competencies to effectively perform their role. There was a strong agreement from participants’ narratives that assuming a new management position requires a mixture of new skills and competencies that will enable new managers to respond to the needs of the public and the organisation. Gauteng province’s healthcare is commonly faced with rapid transitions attributed to technological, policy and consumer-related changes. The complexity of these changes may threaten the organisation’s effectiveness, if not adequately prepared (Dwikat, Arshad & Shariff 2023). The organisation’s ability to thrive through environmental transitions is fully dependent on the competence and skills of its human capital (Dwikat et al 2023). Some of the current study’s participants articulated similar observations when they assumed the role of management, as reported further in the text:

‘I think from public management point of view, skills development would play a role. I think that actual management of the different policies that we have in government needs to be taught more …’ (Female, 40 years old, Chief Radiographer)

‘… I think in basic management, you need to know or be able to deal with strategic and operational planning which is huge in the department. You need skills like computer literacy. Like I said, because without such skills you won’t be able to run a department and leadership skills.’ (Female, 58 years old, Assistant director)

Participants were asked about their perceptions of the management programmes and skills required to succeed in their positions, and they revealed that more knowledge on concepts such as human resource management, strategic planning, policy development and labour relations is required. Participants’ reflections are evidence of this finding:

‘[*W*]e also need courses on business management so to understand the financial aspect of our department and functioning of it. There needs to be at least an outline into training for it and a map on what is expected of you and this is how we do things.’ (Female, 31 years old, Chief Radiographer)

‘… I think training in terms of policies, but I think more about how to implement them and make sure that they are being utilized correctly and everyone knows about the policies… and also, the financial aspects of things.’ (Female, 29 years old, Chief Radiographer)

Ysar et al. (2017) identify continuous on-the-job training as a necessary facilitator for developing relevant management skills and competencies. A well and correctly trained manager should display skills that enable the organisation to prosper through communication, motivation and delegation to others. It is affirmed that the consistent complexity and dynamism of the healthcare environment require strong and effective skills and competencies such as leadership, life-long learning and responsiveness (Sithole 2017). In the current study, it was also mentioned that the element of trust is important in the coaching process because it facilitates a positive and lively exchange of feedback on key performance areas.

### Limitations

The study was conducted only in the public health Radiography departments under GDoH in South Africa. Moreover, the use of online FGDs limited the ability to interpret the non-verbal cues that could have given more insight into the participants’ experiences. The other limitation is that of self-report bias because the researcher was known to participants. This may have had an influence on some participants to provide socially acceptable responses and thus, affecting the accuracy of the collected data.

### Recommendations

Management in the public health sector requires continuous awareness of models of service delivery that will respond to real-life challenges of the service beneficiaries. Guidelines could be developed to improve the managers’ performance and output. Implementation of in-house coaching, mentoring and professional development programmes could aid to enhance the managers’ confidence, skills and expertise. It is also recommended that executive management support programmes be implemented and that more research that focuses on the idea of effective management competency be undertaken in the sphere of Radiography.

## Conclusion

The study explored GDoH radiography managers’ perceptions on management training and skills required to perform their roles optimally. The findings of the current study outline radiography managers’ perceptions and experiences after being appointed into their management position. The public health sector is committed to providing sustainable health and wellness services to the public. Management of the radiography department plays a critical role in modelling and directing resources towards attaining the strategic goals of the organisation. Challenges that radiography managers encounter create certain needs for them during their tenure as managers. These needs include information, emotional support, coaching and self-awareness.

The implementation of structured and mandatory coaching and mentoring programmes for radiography managers could enable the managers to be more skilled, independent, competent and self-reliant. The highlighted perceptions and experiences confirmed the need for the development of relevant, appropriate and contextual support systems. The current study presented lived experiences of the radiography managers who were employed in the public healthcare system in GP. Findings of the study revealed participants’ congruent agreement that transition into the new management position presents challenges and new managers need continuous learning and development opportunities. Furthermore, top management support is necessary to ensure that the transition into a new position is smooth and productive.
